# Genome Sequence of the Plant Growth-Promoting Rhizobacterium Pantoea agglomerans C1

**DOI:** 10.1128/MRA.00828-19

**Published:** 2019-10-31

**Authors:** Francesca Luziatelli, Anna Grazia Ficca, Francesca Melini, Maurizio Ruzzi

**Affiliations:** a Department for Innovation in Biological, Agrofood and Forest Systems, University of Tuscia, Viterbo, Italy; b CREA Research Centre for Food and Nutrition, Rome, Italy; University of Maryland School of Medicine

## Abstract

Pantoea agglomerans strain C1 has plant growth-promoting (PGP) traits and exhibits antimicrobial activity. The genome comprises 4.8 Mb, 4,696 protein-coding sequences, and a G+C content of 55.2%.

## ANNOUNCEMENT

The genus *Pantoea* (family *Enterobacteriaceae*) comprises bacteria that can be isolated from a wide variety of environments and hosts, including soil, water, and plants (1–3). Distinctive strains of *Pantoea* are recognized as plant growth-promoting rhizobacteria (PGPR) and can potentially be used to develop new biofertilizers or biological control agents for sustainable agriculture (4).

*P. agglomerans* strain C1 was isolated from the phyllosphere of Lactuca sativa L. (lettuce) plants treated with vegetal-derived protein hydrolysates (5). It has some interesting plant growth-promoting features, such as the ability to produce indole-3-acetic acid (a plant hormone of the auxin class), and showed antagonistic activity against some phytopathogenic fungi and bacteria (6).

The genomic DNA was extracted from cells grown overnight in Luria broth by using a PureLink genomic DNA minikit (Thermo Fisher Scientific, Italy), as reported elsewhere (6). The genomic library was prepared using a Nextera XT DNA library prep kit and Nextera XT index kit (Illumina, USA). The library fragment size distribution was checked using an Agilent 2100 Bioanalyzer (Agilent Technologies, Santa Clara, CA, USA), and DNA was quantified using a Qubit 2.0 fluorometer and the Qubit double-stranded DNA (dsDNA) high-sensitivity (HS) assay kit (Thermo Fisher Scientific). The whole-genome sequencing of strain C1 was performed at Bio-Fab Research S.r.l. (Rome, Italy) using the MiSeq platform (paired-end 2 × 300 bp) with the MiSeq reagent kit v3 (Illumina). In total, 7,525,566 reads were obtained, achieving about 150-fold genomic coverage. Read quality was checked using FastQC 0.11.5 (7), whereas adapter and low-quality sequences were trimmed using Trimmomatic 0.39 (8). Default parameters were used for all software unless otherwise noted. The resulting reads were assembled into contigs using the A5-MiSeq assembly pipeline (version 20160826) (9), and the quality of the assembly was assessed using QUAST version 5.0.2 (10). The Mauve tool (11) was used to align and order the assembled contigs using Pantoea agglomerans strain C410P1 (GenBank accession number CP016889) as a reference.

The genome assembly produced 22 contigs, with an *N*_50_ value of 645,999 bp, and the largest contig was 2,247,784 bp. The genome of *P. agglomerans* C1 was 4,846,925 bp, with a G+C content of 55.2%, which falls within the genome size (4.5 to 6.3 Mb) and G+C content (52% to 55%) of other strains belonging to the same genus. The RAST*tk* (12, 13) and PATRIC (14) programs were used for genome annotation, with default parameters and using the *Enterobacteriales* data set as a reference. No plasmid was detected in the genome by using Plasmid Finder (15). *P. agglomerans* C1 has 4,696 protein-encoding genes (PEGs), as predicted using GLIMMER 3 (16), of which 3,784 (81%) have a predicted function assigned using the GenDB annotation pipeline (17), and 1,383 (30%) and 975 (20%) PEGs were assigned to an Enzyme Commission (EC) and Gene Ontology (GO) functional category, respectively. Furthermore, a total of 70 tRNA-coding genes and 12 rRNA genes were predicted in the chromosome sequence. Genes potentially involved in the promotion of plant growth were also carefully mined. This analysis allowed the identification of genes for phosphate solubilization and the production of phytohormones, pest/disease-suppressing γ-aminobutyric acid (GABA), siderophores, organic acids (e.g., gluconic acid), and volatile compounds that influence plant growth and increase plant resistance against pathogens.

Manual analysis revealed that the C1 genome carries cluster operons encoding putative arsenic (As) and copper (Cu) resistance-related proteins, as well as genes for cadmium (Cd) tolerance and oxidative stress response. Furthermore, two distinct large intact prophage regions, named prophage_1 and prophage_2, were examined using the PHAge Search Tool (PHAST) (18). The prophage_1 region has a size of 39.70 kb, with a G+C content of 52.51%, and 62.22% similarity to PHAGE_Erwini_ENT90_NC_019932, encompassing 41 out of 45 open reading frames (ORFs) present in the region that matches the phage protein database. The prophage_2 region was 30.1 kb, with a G+C content of 52.50%, and 55.81% similarity to PHAGE_Salmon_RE_2010_NC_01948, with 32 out of 45 ORFs that match the phage protein database.

### Data availability.

The genome sequence of *P. agglomerans* C1 is available under NCBI BioProject accession number PRJNA523737, with Sequence Read Archive (SRA) accession number SRP212904.
